# Peeling of Flexible Laminates—Determination of Interlayer Adhesion of Backsheet Laminates Used for Photovoltaic Modules

**DOI:** 10.3390/ma15093294

**Published:** 2022-05-04

**Authors:** Gernot Oreski, Gerald Pinter

**Affiliations:** 1 Polymer Competence Center Leoben GmbH, 8700 Leoben, Austria; 2 Department Polymer Engineering and Science, Montanuniversitaet Leoben, 8700 Leoben, Austria; gerald.pinter@unileoben.ac.at

**Keywords:** peel testing, flexible laminates, photovoltaic backsheets

## Abstract

Delamination is one of the most critical failure modes of a PV module during service lifetime. Delamination within a backsheet primarily imposes a safety risk, but may also accelerate various other PV module degradation modes. The main aim of this paper is to present a peel test set-up, which is more practical in sample preparation and execution than the width-tapered cantilever beam test and overcomes some issues of standard peel tests like the influence of sample geometry and energy dissipation through deformation on the peel test results. The best results with respect to accuracy and effort were achieved by using a 180° peel geometry where an additional adhesive tape is applied to the peel arm in order to avoid plastic deformation or breakage. The additional support of the adhesive tape leads to comparable peel strength values without any influence of the plastic deformation behavior of the peel arms with different thickness.

## 1. Introduction

The general architecture of wafer-based crystalline silicone photovoltaic (PV) modules has been developed since more than 40 years ago within the Flat-Plate Solar Array Project and has not significantly changed since then [1]. A modern PV module consists of a number of interconnected solar cells encapsulated into a single, long-lasting, stable multi-material composite. In most cases, surrounded by a frame providing the necessary structural support and usually needed for module mounting, the actual module has a layered encapsulation structure designed to protect the solar cells and their interconnecting wires from the harsh environment in which they are typically used. The primary purpose of this layout is mechanical and environmental protection and electrical safety [2,3]. A typical PV module thus includes a front cover, usually made of glass, a polymeric encapsulation material, and a backsheet. In general, the backsheet has to fulfil similar requirements as the front sheet, which includes weathering protection of the inner layers and electrical insulation of the module. Additionally, the backsheet needs to act as a barrier against water/water vapor ingress [4,5]. To address the manifold requirements polymeric multi-layer films are usually used. The first generation of backsheets were developed in the 1980s, consisting of polyvinyl fluoride (PVF) on the outsides (inner and outer layer) and polyethylene terephthalate (PET) as middle layer. Also, other fluoropolymers, such as polyvinylidene fluoride (PVDF), are used as outer layers [4,6,7]. Due to the general price pressure, a variety of different polymer materials and new backsheet designs have been developed and introduced into the market. These range from fluoropolymer-based or fluoropolymer-free backsheets, to laminates, co-extruded films, monolayer and coated monolayer films. The primary materials used are PET, polyamide (PA), polyethylene (PE), and polypropylene (PP) as core layers and PET, PE, PA, and PP as inner or outer layers [8,9,10].

Delamination of one or more layers is one of the most critical failure modes of a PV module during its service lifetime [11,12,13], so its root causes have been investigated thoroughly [5,14,15,16]. Delamination within a backsheet primarily imposes a safety risk due to failing wet leakage insulation [7,17]. Additionally, backsheet delamination may accelerate various other PV module degradation modes such as corrosion, potential induced degradation (PID) or polymer hydrolysis by providing gateways of moisture ingress into the modules [13,18], which could result in a performance loss over time.

Nevertheless, a proper evaluation of adhesion within a backsheet laminate is still a challenge, especially for the many different new materials that have been introduced into the market. Different adhesion tests have been reported in the scientific literature, from simply measuring the peel force using T-Peel tests directly on the laminates [5] to more complex test setups based on fracture mechanics principles such as the width-tapered cantilever beam test proposed by Tracy et al. [19,20], which can be applied to PV modules directly.

**Figure 1:**
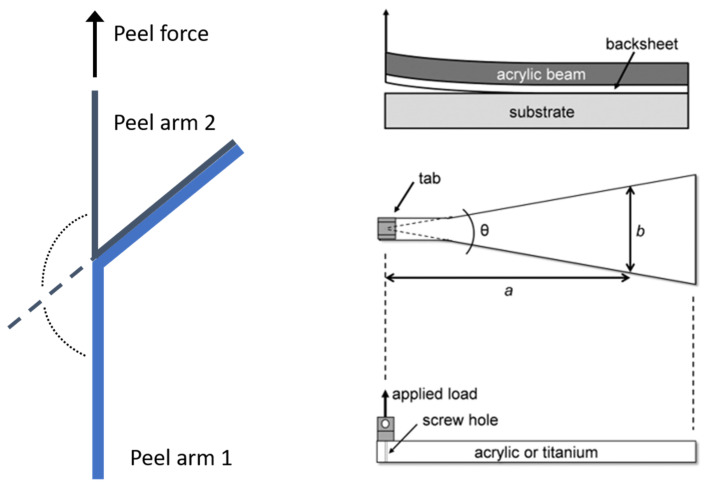
Configuration of T-Peel (**left**, adapted with permission from Ref. [25]. 2008, Elsevier) and width-tapered beam test (**right**, reprinted with permission from Ref [19]. 2016, John Wiley and Sons).

T-peels are a form of tensile peel separation strength measurement of two flexible materials that have been bonded together such that the entire setup forms a “T” shape during testing (see Figure 1 left). Whereas T-Peel tests are comparatively simple with respect to sample preparation and experimental procedure, it is well described in the scientific literature [21,22,23,24] that an experimental determination of the peel force in N per unit width is not sufficient for describing the characteristics of the adhesion within a flexible, polymeric laminate. Generally, the measured peel force values are dependent on several factors like test geometry, peel angle, test rate, and temperature. Furthermore, the energy dissipation, trough tensile deformation, and bending of the peel arm, which consists of the stored strain energy and plastic or viscoelastic energy dissipation, affect how much of the external work was actually available for debonding the laminate [21]. Finite element analysis showed that during a peel test energy is dissipated in a small region near the interfacial crack tip. Singular strains develop in this region, along with inelastic bending of the peel arm [23]. Therefore, out of this energy balance consideration, the measured peel force P is a function of the interfacial adhesive fracture energy, the peel angle, and the viscoelastic energy dissipation. An additional problem that was reported was the fracture of the peel arm before delamination started [5].

By comparison, using the width-tapered cantilever beam test (see Figure 1, right), direct access to the adhesion energy is obtained, which is less dependent on the viscoelastic properties of the polymeric material, and so more useful for measuring differences or changes in adhesive strength. However, compared to T-Peel tests, sample preparation and test setup are elaborate. A width-tapered cantilever beam made of acrylic or titanium has to be bonded directly to the layer of interest. In the case of a PV module this would be the outer layer of the backsheet. When the beam is loaded at its apex, delamination starts at the weakest interface and progresses with further loading, sometimes also changing the interfaces during the test. Even though an IEC test for the width-tapered cantilever beam test is in development, the method is not widely used in the PV industry for quality control.

The main aim of this paper is to present a simple peel test setup, which overcomes issues from the T-Peel test and is more practical in sample preparation and execution than the width-tapered cantilever beam test.

Hence, an adapted set-up using a 180° peel geometry (derived from ISO 8510-1 [26]) has been evaluated, where an additional adhesive tape is applied to the peel arm in order to avoid plastic deformation or breakage. Also, material-specific influencing factors like anisotropic mechanical properties of the peel arm on peel strength results were investigated. Finally, different evaluation methods (peel strength and adhesive fracture energy) have been compared.

## 2. Materials and Methods

In this work two different multi-layer films, which are used as PV backsheets, were investigated. The films consist of PA outer layers which are laminated to a PET core layer. Both films have the same layer composition except for the thickness of both outer PA layers, which is 25 µm for Laminate 1 and 45 µm for Laminate 2, respectively. To investigate the layer adhesion, 180° peel tests were performed according to ISO 8510-1: Part 2 [26]. For this purpose, the backsheets were laminated to glass using a standard solar cell encapsulation film made of ethylene vinyl acetate (EVA) at standard processing conditions (20 min at 150 °C).

In order to investigate the influence of orientation within the PA peel arm, peel test specimens cut in machine direction (MD) and transverse direction (TD) were investigated. For the delamination tests, rectangular specimens with two different geometries were prepared by cutting the peel arm with a scalpel:

- Method A: Standard approach
  ○ Sample width: 10 mm
- Method B: Engineering approach
  ○ Sample width: 20 mm
  ○ Additional adhesive tape is applied to the PA peel arm in order to avoid full-field plastic deformation or breakage

The peel tests were carried out with a screw-driven universal test machine (Zwick Z010 Allround-Line, Zwick, Ulm, Germany) at a test speed of 50 mm/min at a temperature of 23 °C.

In addition, tensile tests on the PA peel arms at both thicknesses were done to describe the elastic and plastic deformation behavior. In order to investigate the influence of orientation within the PA peel arm, specimens cut in MD and TD were investigated. Tensile tests were carried out with a screw-driven universal test machine (Zwick Z010 Allround-Line, Zwick, Ulm, Germany) at 23 °C according to EN ISO 527-3 [27]. Rectangular specimens of 100 mm in length and 10 mm in width have been used. The test speed was 50 mm/min. From a total of at least ten specimens for each test series, average numbers for elastic modulus (E), yield strength (σ_y_), stress at break (σ_b_), and strain at break (ε_b_) were deduced.

## 3. Results

**Figure 2:**
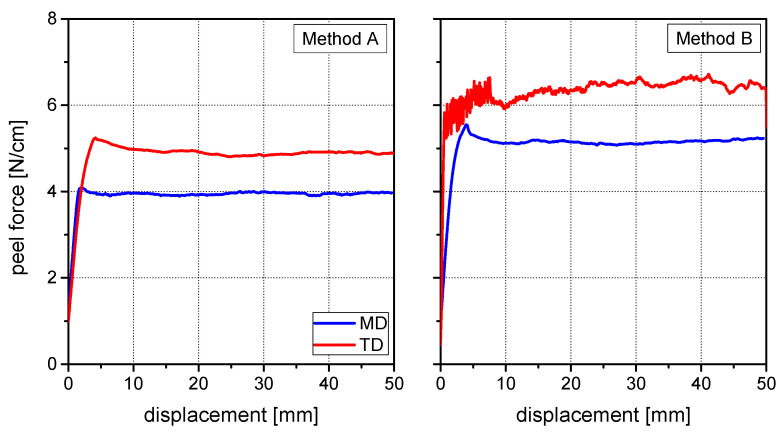
Peel curves of Laminate 1 in machine and transverse direction: Method A (**left**) and Method B (**right**).

**Figure 3:**
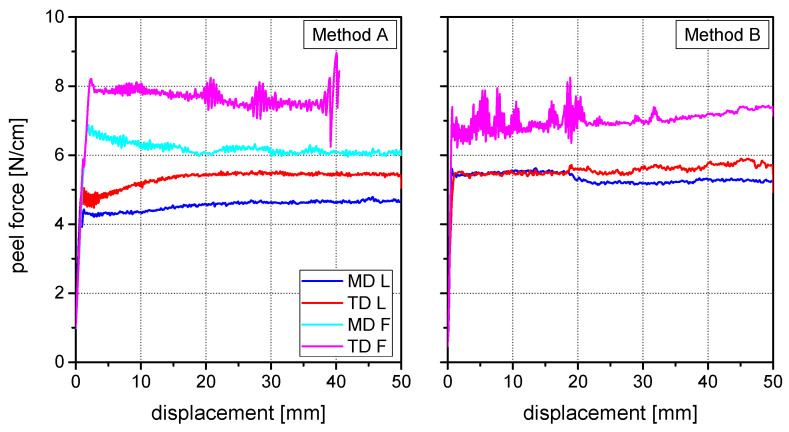
Peel curves of Laminate 2 in machine and transverse direction: Method A (**left**) and Method B (**right**).

**Figure 4:**
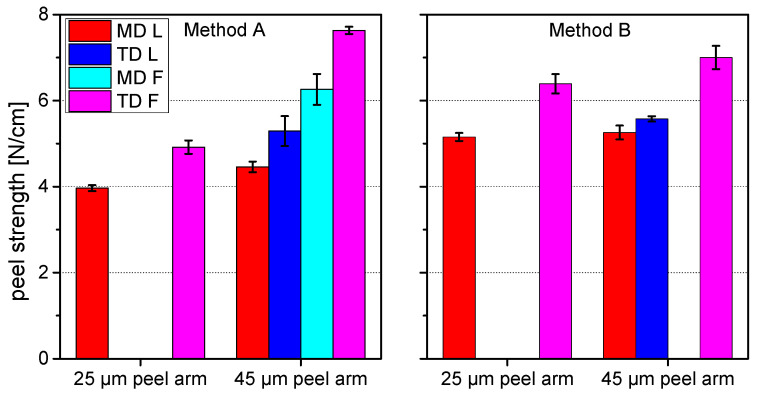
Peel strength values obtained from Method A (**left**) and Method B (**right**); values have been clustered with respect to the observed deformation mode, missing bars indicate that the specific deformation mode has not occurred.

Figure 2 and Figure 3 show the peel curves of both laminates for methods A and B in both machine and transversal direction. The obtained peel strength values are depicted in Figure 4. At both specimen geometries a significant difference between peeling in machine direction and peeling in transverse direction was observable. The peel forces measured in transverse direction were significantly higher than the peel forces measured in machine direction. Moreover, method B with the additional adhesive tape leaded to higher peel force values than method A.

More interestingly, for each case different deformation modes were observed. Regarding method A, the PA peel arms showed linear elongation in both measured directions. In the machine direction this effect was quite limited, resulting in a peel arm elongation between 2 and 5 mm and nearly no visible necking. In the transverse direction however, strong peel arm elongation between 25 and 50 mm as well as significant necking was observed. The effect of applying an additional adhesive tape is, along with other benefits, the restraint of this elongation. Also, in the case of method B two different deformation modes were observed. During measurement in the machine direction, no deformation of the peel arm and a linear front crack was observed. In the transverse direction on the contrary, the peel arm showed strong plastic deformation near the crack front. Furthermore, delamination between the peel arm and the additional adhesive tape was also observed. When compared to the machine direction, in the transverse direction the crack front was curved.

Two fundamental conclusions can be drawn from the measured results and the investigation of the deformation modes:

(1) Higher plastic deformation of the peel arm—globally (method A) or locally (method B)—is associated with higher peel forces.
(2) The reinforcement of the thin peel arm with an additional adhesive tape leads to higher peel force values.

Laminate 2 has the same composition as Laminate 1, with exception of the thicker outer PA layer, which is 45 µm compared to 25 µm. The peel tests revealed a more complex deformation behavior than for the Laminate 1, although in general, similar effects with some deviations were observable. At both specimen geometries a significant difference between peeling in the machine direction and peeling in the transverse direction was observable. The peel forces measured in the transverse direction were significantly higher than the peel forces measured in the machine direction.

As before, for each case different deformation modes were observed, but this time also within the same method and load direction. Consequently, for method A four different peel force values were obtained with a high reproducibility. For each load direction low (L), small-field, or full-field (F) plastic deformation mechanisms were observed. In the machine direction half of the investigated specimens exhibited a linear peel arm elongation below 1 mm with limited necking and a linear crack front. The other half of the tested specimens showed higher linear elongation values and significant necking, but more important, full-field plastic deformation which resulted in a distortion of the peel arm. The same two deformation modes were observed in the transverse direction, but in both cases leading to higher plastic deformation than in the machine direction.

Regarding method B, in the machine direction only one deformation mode was observed after peel testing, with no deformation of the peel arm and a linear front crack. In the transverse direction, however, localized (L), small-field, as well as full-field (F) plastic deformation mechanisms were observed. About half of the specimen showed no deformation of the peel arm and a linear front crack. The second half of the specimen exhibited a slight plastic deformation near the front crack. Furthermore, delamination between the peel arm and the additional adhesive tape was also observed. Additionally, compared to the machine direction, in transverse direction the front crack was curved. Supposedly this effect was smaller than for the 25 µm peel arm due to the higher peel arm thickness.

**Figure 5:**
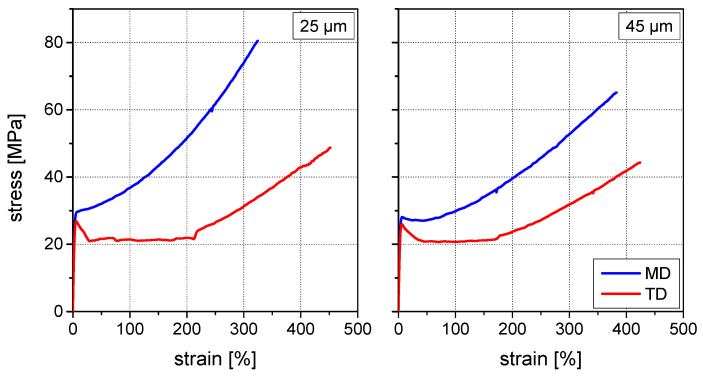
Stress–strain curves of PA peel arms in MD and TD: 25 µm thickness (**left**) and 45 µm thickness (**right**).

To explain the different phenomena, tensile tests of the peel arm in both directions were carried out. The stress–strain curves of the 25 µm and 45 µm thick PA peel arms for machine and transverse direction are depicted in Figure 5. The direction of measurement reveals significant differences in the mechanical properties for both film thicknesses.

In machine direction, the stress–strain curve of the 25 µm film shows a ductile material behavior with more or less bilinear characteristic with an elastic modulus of 970 ± 40 MPa and a yield strength value of 29.3 ± 1.3 MPa. In machine direction, the 45 µm film forms a more pronounced yield point than the 25 µm film. Slightly lower elastic modulus value of 930 ± 40 MPa and yield strength value of 28.3 ± 1.0 MPa were obtained.

Directly after the yield point strong strain hardening can be observed for both film thicknesses. This behavior can be attributed to strong orientation of the molecular chains along the machine direction during film extrusion, but due to the higher film thickness this effect is less pronounced for the 45 µm film than for the 25 µm film.

In transverse direction however, a totally different deformation behavior was observed. Elastic modulus and yield strength values were slightly lower but comparable to the values measured in machine direction (see Table 1). Due to the tensile load transverse to the molecular orientation the film strong plastic deformation was observed after exceeding the yield point at a more or less constant stress level, where the molecules are re-oriented in the load direction. Strain hardening is not observed before approximately 200% elongation, where most of the molecules are re-oriented in the load direction. Generally, due to the higher film thickness lower orientation effects can be expected, which is expressed in lower modulus and yield strength values.

In the following, the influence of the mechanical behavior of both peel arms is discussed.

Laminate 1: The stress–strain curves in machine or in transverse direction explain the differences in the deformation behavior of the peel arm during peel testing. In the machine direction no or only limited elongation effects were observed due to the high molecular orientation and the resulting strain hardening. In the transverse direction, strong plastic deformations of the peel arm result from the re-orientation process of the chain molecules after exceeding the yield strength. In the case of method A, a full-field elongation of the peel arm was observed. In the case of method B, where a full-field plastic deformation of the peel arm is inhibited, plastic deformation was localized at the crack tip only, where a local stress concentration is expected due to the test geometry [21,23,24].

Laminate 2: The deformation modes of the peel arm during peel test for machine and transverse directions as well as for methods A and B are comparable to the modes of Laminate 1. The appearance of localized (L), small-field as well as full-field (F) plastic deformation mechanisms within the same test set-up may be explained by the scattering of the yield strength values. The plastic deformation of the peel arm during peel testing is strongly depending on the local stress concentration in the peel arm at the crack tip [1,2,3]. Deformation can be expected if the local stress at the crack tip exceeds the yield strength of the full-field plastic material. A verification of this assumption can be done by estimating the local stress at the crack tip using the IC Peel test protocol, which was developed at the Imperial College London [22,25,28,29].

## 4. Discussion

### 4.1. Determination of Adhesive Fracture Energy

The best way to assess adhesive strength would be calculation of the so-called adhesive fracture energy according to Moore and Williams [22,25,28]. This energy-based fracture mechanics approach takes specimen geometry (e.g., backing foil) and testing parameters into account and provides reliable, absolute adhesion strength values. But, to calculate the adhesive fracture energy two experiments are required. First a Peel test has to be carried out to measure the peel force. Second a tensile test of each peel arm has to be performed. The tensile properties are necessary to calculate the corrections due to plastic bending deformation. With the calculation of the adhesive fracture energy, the anisotropy effects due to molecular orientation in the peel arms are also considered by the mechanical properties of the peel arm. The adhesive fracture energy *G_C_* can be calculated according to Equation (1):

(1) $$G_{C}=\frac{1}{b}\times \left(\frac{dU_{ext}}{da}-\frac{dU_{S}}{da}-\frac{dU_{dt}}{da}-\frac{dU_{db}}{da}\right),$$

using external work *U_ext_*, stored, reversible tensile strain energy *U_s_*, dissipated energy through irreversible tensile (*U_dt_*) and bending (*U_db_*) deformation of peel arm, specimen width *b* and displacement of peel front (*da*) [22,28,30].

The calculation of the adhesive fracture energy was done using the ICPeel (2006) protocol [29]. Even though the protocol was set up for flexible laminates, the estimation of the high strain modulus E_2_, which is needed for the calculations, proved to be challenging because of the high plastic deformation of the peel arms. The power law fit and the digitized approximation were found to not be suitable. The bilinear approach only worked for the 25 µm peel arm in machine direction, where a value for α = 0.0037 was found. A is calculated as ratio between the high strain modulus E_2_ and the elastic modules E [22,28]. For all other cases a value for α of 0.0001 was assumed. For the adhesive layer a thickness of 8 µm and a modulus value of 5 MPa was estimated.

Table 2 summarizes the calculated values for adhesive fracture energy *G_C_*, the plastic work in bending *G_D_*, the correction *G_D_/G_tot_* (input enery corrected for stored strain energy and tensile dissipations on the peel arm) and the calculated maximum stress for the damage zone *σ_max_* (*o*).

The tensile corrections were found to be negligible and bending of peel arm is dominating energy dissipation. As suspected, the high plastic deformations of the peel arms are not considered accurately, otherwise the adhesive fracture energy values would have been in a similar range for all peel arm thicknesses and measurement directions. Also, the size of the correction is between 27 and 49%, so the error in determination of adhesive fracture energy may become significant. Interestingly, the calculated maximum stress for the damage zone is in the region of the yield stress.

In summary, the determination of adhesive fracture energy is not practical for evaluation of adhesive strength of investigated laminates, where the peel arms show high plastic deformation.

### 4.2. Recommenations for Peel Testing of PV Backsheets

The most meaningful results should be expected from a 180° peel test with method B. The additional support of the adhesive tape not only prevents thin peel arms from breaking before peeling [4,5], but also restrains full-field plastic deformations of the peel arm. The only requirement for the appropriate assessment of the adhesive strength is an evaluation of the local peel arm deformation mode at the crack front. Different deformation modes may occur depending on the yield strength of the peel arm. The only specimen with no visible plastic deformation, a linear front crack, and no visible delamination of the adhesive tape from the peel arm have to be considered. This conclusion is confirmed by the good consistency of the peel force values of laminate 1 (MD) and laminate 2 (MD and TD), where no plastic deformation or necking was observable (see Figure 3).

When conducting peel tests without adhesive tapes, at least the anisotropy of the mechanical properties of the thin peel arms has to be considered. Therefore, peel tests have to be conducted in machine as well as transverse directions, and isotropic peel force values have to be derived. The mean values of the isotropic peel forces of method A is 4.4 ± 0.5 N/cm for Laminate 1 and 5.9 ± 1.1 N/cm for Laminate 2. This significant difference can be assigned to the different film thicknesses and therefore different plastic deformation behavior of the PA peel arm. The higher film thickness resulted in a higher plastic deformation of the peel arms and therefore higher peel force values.

## 5. Conclusions

Delamination of one or more layers is one of the most critical failure modes of a PV module during service lifetime. A proper evaluation of adhesion within a backsheet laminate is still a challenge, as different adhesion tests have been reported in the scientific literature, from simply measuring the peel force using T-Peel tests to more complex test set ups based on fracture mechanics principles such as the width-tapered cantilever beam tests. The main aim of this paper was to present a peel test, which overcomes the issues from T-Peel test and is more practical in sample preparation and execution than the width-tapered cantilever beam test.

The main objective was to make the adhesion values for different laminate constructions more comparable using a simple peel test geometry. Hence, an adapted set-up using a 180° peel geometry has been evaluated, where an additional adhesive tape is applied to the peel arm in order to avoid plastic deformation or breakage.

The investigation showed the measured peel force values are dependent on several factors, such as load direction and peel arm thickness, since energy dissipation trough tensile deformation and bending of the peel arm is not considered. Not even the fracture mechanics approach of calculation of the adhesive fracture energy delivers satisfactory results which are independent from sample geometry. This is due to the high plastic deformation behavior of the polyamide peel arm, which makes it difficult to determine calculation parameters for the adhesion fracture energy.

The best results by means of accuracy and effort have been achieved using the “engineering approach”, a 180° peel test with an additional polyester adhesive tape applied to the peel arm. The additional support provided by the tape prevented fracture and full-field plastic deformation of the peel arms, which subsequently resulted in comparable, isotropic peel strengths with no effect on the plastic deformation behavior of the peel arms of different thicknesses. The proposed test setup is more practical in sample preparation and execution than the width-tapered cantilever beam test and overcomes some issues typical for influencing factors of standard tensile peel test results such as sample geometry or energy dissipation through deformation.

## Figures and Tables

**Table 1:** Tensile test results for both film thicknesses in machine direction (MD) and transverse direction (TD).

Thickness	Elastic Modulus E		Yield Stress σ_y_	
[µm]	[MPa]		[MPa]	
	MD	TD	MD	TD
25	966 ± 43	906 ± 33	29.3 ± 1.3	27.0 ± 1.1
45	934 ± 35	871 ± 13	28.4 ± 1.3	26.3 ± 1.1

**Table 2:** Results from ICPeel (2006) test protocol, with adhesive fracture energy *G_C_*, the plastic work in bending *G_D_*, the input energy corrected for stored strain energy and tensile dissipations on the peel arm *G_tot_*, and calculated maximum stress for the damage zone *σ_max_* (*o*). Values have been obtained in machine (MD) and transversal (TD) direction, also the appearance of localized (L), small-field as well as full-field (F) plastic deformation mechanisms has been documented.

Thickness	Direction	Deformation	*G_C_*	*G_D_*	*G_tot_*	Correction	*σ_max_* (*o*)
[µm]	-	-	[J/m^2^]	[J/m^2^]	[J/m^2^]	[%]	[MPa]
25	MD	S	535	268	803	33	26
25	TD	F	716	269	985	27	30
45	MD	S	465	437	902	49	24
45	TD	S	610	453	1064	43	28
45	MD	F	764	501	1265	40	31
45	TD	F	1013	515	1527	34	35

## Data Availability

The data presented in this study are available on request from the corresponding author. The data are not publicly available due to confidentiality.
